# The Association between Subclinical Atherosclerosis and Uterine Fibroids

**DOI:** 10.1371/journal.pone.0057089

**Published:** 2013-02-22

**Authors:** Yuan He, Qiang Zeng, Xiaohui Li, Baohua Liu, Peiyu Wang

**Affiliations:** 1 International Medical Center, Chinese People's Liberation Army General Hospital, Beijing, China; 2 Department of Nutrition and Food Hygiene, School of Public Health, Peking University Health Science Center, Beijing, China; 3 Department of Social Medicine and Health Education, School of Public Health, Peking University Health Science Center, Beijing, China; Sapienza University of Rome, Italy

## Abstract

**Objective(s):**

To explore the atherogenic hypothesis of uterine fibroids among Chinese women.

**Methods:**

In a case-control study, 335 patients confirmed by ultrasound or hysterectomy surgery and 539 controls were enrolled between October 1, 2009 and April 1, 2012. Unconditional logistic regressions were used to calculate the odds ratios (ORs) for the associations of subclinical atherogenic and cardiovascular risk parameters with uterine fibroids in the overall case group and hysterectomy-confirmed case group, respectively.

**Results:**

Higher level of ankle-brachial index (ABI) was independently associated with increased odds of uterine fibroids. The odds of UF among women in the highest tertile of ABI were 1.88 times higher (95%CI: 1.17, 3.02, P_trend_ = 0.008) compared to those in the lowest tertile. The serum concentration of homocysteine was inversely related to fibroids (middle vs. low: OR 0.56, 95%CI: 0.36, 0.85; high vs. low: OR 0.50, 95% CI: 0.32, 0.79; P_trend_ = 0.002). In the hysterectomy-confirmed group, an inverse association was suggested between high-density lipoprotein cholesterol (HDL-C) and fibroids (OR 0.46, 95% CI: 0.25, 0.84, P_trend_ = 0.014). Moreover, the effect of homocysteine concentration was not observed in this group.

**Conclusion(s):**

These findings suggest that women with uterine fibroids might have an increased risk of subclinical atherosclerosis.

## Introduction

Uterine fibroids (UF) are common tumors of the uterus smooth muscle and represent a primary indication of hysterectomy [1], [2]. It has been reported that 30–70% of women have UF in their premenopausal years based on different diagnoses [3], [4]. In China, approximately one in four adult woman have ultrasound-confirmed UF by the age of 50 (unpublished data), a rate that is similar to that found in American White women [5], [6]. Symptomatic UF can cause pelvic pain, low back pain, irritable bowel, heavy menstrual bleeding and even premature labour and infertility [2], [7]. Although UF are rarely associated with mortality, they usually cause significantly increased health care costs and negative health experiences [8], [9].

Major studies have suggested that UF represent a hormone-dependent disease [10], [11]. Established risk factors include age, African-American heritage, early age of menarche and nulliparity [10], [12]. African-American women have been reported to have 2–3 times the risk of UF than White women [6], [13], a difference that could not be explained by ethnic differences in hormone levels and other known risks [3], [6]. In comparison with White women, Black women have a higher prevalence of hypertension, diabetes and obesity [14], which are independently associated with UF [12], [15], [16]. These factors are also common cardiovascular risks and might be related to atherosclerosis [17]. The shared patterns support the hypothesis that the development of UF and the development of atheromatous plaques share a common biological mechanism [15]. In addition, proliferating smooth muscle cells of a monoclonal origin are critical in the formation of both UF and atheromatous plaques [18], and cells from these two conditions behave identically in culture [19].

Pulse wave velocity (PWV) and ankle-brachial index (ABI) are sensitive and noninvasive tools to evaluate the risk of early atherosclerosis [20], [21]. Powerful predictors of cardiovascular diseases, including serum C-reactive protein (CRP), homocysteine, folate and vitamin B12, have been validated to accelerate or inhibit the process of atherosclerosis in animal and epidemiological studies [22], [23], [24]. If the growth of UF depends on atherogenesis hypothesis, then an analogous association of these factors with UF should be observed. However, no evidence of this association has been reported to date. Preponderance of UF literature is based on data from Western countries [15], [25], [26], the relevant evidence of Asian women is very limited. To further understand the atherogenesis hypothesis, we analyzed data from a large sample of Chinese women in a case-control study.

## Materials and Methods

### Participants

This study was conducted in the International Medical Center of the People's Liberation Army General Hospital (PLA General Hospital) in Beijing between October 1, 2009 and April 1, 2012. Study subjects were premenopausal women lived in the urban regions of North China who participated in regular health examinations on the purpose of early detection and treatment of gynecological diseases. Most of the subjects were from government agencies and local companies, and were characterized by a relative higher socioeconomic status. There were about 2000 women came for health examinations in this center annually, of whom 10–15 percent were diagnosed as UF cases. Because UF may shrink or disappear by menopause, we excluded postmenopausal subjects who had been without a menstrual cycle in the past 12 months before the date of the examination as reported in the medical records. The following conditions were also considered as eligibility criteria for both groups: 1) Women had no acute, hormonal or neoplastic conditions, 2) were within the age range of 35 to 55 years old, and 3) were not pregnant or breastfeeding during the study period.

The case group consisted of 335 women (mean age: 48.10±4.33; age range: 35–55) with ultrasound or surgically confirmed UF. Cases of UF were confirmed consecutively by professional gynecologists based on transabdominal or transvaginal ultrasound diagnoses during the study period. Transvaginal ultrasound was used in patients who had small UF or UF that were located in the posterior uterus, which cannot be detected accurately through transabdominal ultrasound. Women who underwent hysterectomy surgeries for the indications of UF were also enrolled because they are generally categorized as severe UF. After the study was introduced to all subjects, initial enrollment involved 443 eligible cases with a response rate of about 90 percent. The participating cases didn’t differ from the declined cases in any sense. We excluded 25 cases because their sonogram reports included any phrases such as “questionable UF” or “possible UF”. We also excluded 63 women who are less than 35 years old or more than 55 years old. Additionally exclusion was 20 case women with a history of gynecologic or breast cancer. Of the final 335 premenopausal women, the majority (>90 percent) were diagnosed by transabdominal ultrasound. The cases diagnosed by ultrasound were slightly younger than the cases diagnosed through hysterectomy.

The control group consisted of 539 women (mean age: 45.97±5.28; age range: 35–55) with intact uteri. Controls were women who had regular health examinations, including a transabdominal ultrasound screening by the same gynecologists as the case subjects in the same hospital with a comparable catchment area. The control subjects were enrolled if they had not any abnormal gynecological condition, a confirmed or suspected history of UF. We excluded controls with self-reports of “no UF” because undiagnosed UF are common [4]. Other inclusion criteria pertaining to cases were also applied to controls: urban residents in North China, premenopausal status, no history of acute, hormonal or neoplastic conditions, aged 35–55 years old, and not pregnant or breastfeeding. A total of 2381 women were screened, and 539 eligible controls (23 percent) were identified. Of these, 30 percent were admitted for metabolic disorders, 25 percent had stomach and digestive illnesses, 10 percent had osteoporosis, 10 percent had stone conditions, 10 percent had eye disorders and 15 percent had other illnesses, such as ear, nose and throat or dental disorders. The participating controls didn’t differ from the declined controls regarding the age and socioeconomic aspects.

### Ethics Statement

This study was approved by the Institutional Review Board of the People's Liberation Army General Hospital and was defined according to the Declaration of Helsinki. All subjects signed informed consent forms. Confidentiality of the subjects was maintained at all times.

### Clinical and Laboratory Assessment

All the atherosclerotic risk factors were measured at the time of the health examination between October 1, 2009 and April 1, 2012. Height, waist and hip circumference were measured to an accuracy of 0.1 cm, and body weight was measured to an accuracy of 0.1 kg. Systolic blood pressure (SBP) and diastolic blood pressure (DBP) were measured on the right arm with an electronic sphygmomanometer after the patient was seated for five minutes. PWV and ABI were measured using a non-invasive vascular screening device (model BP-203 RPE II; Colin Medical Technology, Komaki, Japan) after a five-minute rest in a supine position. PWV (cm/s) was defined as the distance between two distinct points (cm) divided by the pulse wave transit time (s). The pulse volumes in the brachial and posterior tibial arteries were recorded. ABI was measured while the subjects were in the supine position. The maximum blood pressure values in the brachial artery, posterior tibial artery and dorsalis pedis artery were measured. ABI was calculated as the posterior tibial artery divided by the brachial artery on the right and left sides. The average value of the right and left sides was used for analysis. The anthropometric measurements were conducted by trained study personnel.

Fasting blood samples were collected in the early morning for the measurement of total cholesterol (TC), triglycerides (TG), high-density lipoprotein cholesterol (HDL-C), apolipoprotein A1 (ApoA1), apolipoprotein B (ApoB), apolipoprotein E (ApoE), lipoprotein(a) and serum glucose for the overall group. Low-density lipoprotein cholesterol (LDL-C) was calculated using the Friedewald equation (LDL-C (mmol/L) = TC-[HDL-C+TG/2.2]). Additionally, serum levels of C-reactive protein (CRP), homocysteine (Hcy), folate (Fol), and vitamin B12 (Vit B12) were measured in a subgroup consisted of 232 cases and 366 controls, with the exception of the 276 subjects who refused these measurements (31 percent and 32 percent of eligible cases and controls, respectively). All biochemical measurements were performed in the clinical laboratory of the PLA General Hospital using standard techniques.

### Other Data Collection

Information on each woman’s reproductive history was abstracted from her medical records, including the age at menarche, number of pregnancies, number of live births, age at last birth and menopausal status. Abstractions were conducted by two independent assistants and less than 0.01% of the data was found to be inaccurately. BMI was calculated as weight/height^2^. The waist-to-hip ratio (WHR) was calculated as the waist circumference divided by the hip circumference. The index of dyslipidemia was calculated as TG/HDL-C, and index of atherogenicity was calculated as LDL-C/HDL-C.

### Statistical Analyses

Descriptive statistics for all variables were computed. Continuous variables were expressed as means ± standard deviation (SD) and compared using the *t*-test for independent groups. Categorical variables were expressed as frequencies and percentages, and percentages were compared with the chi-square test. The variables were divided into tertiles based on the values of control group, and the lowest tertile was used as the reference group. First, separate multivariate logistic regressions were used to evaluate the atherogenic and cardiovascular risk parameters that were independently related to UF while adjusting for confounding factors including age, BMI (except BMI-UF association), age at menarche, gravity and age at last birth, as specified in the tables. Because the serum concentrations of folate and vitamin B12 could influence the metabolism of homocysteine, we additionally controlled for these two nutrients to assess the association between homocysteine and UF besides other common confounders. The results for the overall case group and for the hysterectomy-confirmed case group were presented respectively. Tests for trend were performed by fitting the median of the variables for each tertile as a continuous variable in the models. Next, complete regressions included all associated putative risk factors were built using a stepwise selection procedure based on a likelihood ratio test to determine the individual contribution to UF after adjusting potential confounders. Tests for multiplicative interactions between these putative risk factors were performed by entering each product term into the multivariate model with a Wald statistic. The statistical analyses were performed using SPSS 16.0 (SPSS Inc., Chicago, Ill., USA). Statistical significance was considered at p<0.05.

## Results

We first compared the baseline characteristics of women with UF and controls (Table 1). 57.3% of the cases were ultrasound-confirmed UF, while the remaining cases had undergone hysterectomy (42.7%). With increasing age, a positive association was observed between age and the presence of UF (Table 1). Women aged 40–49 had nearly 3-fold increased odds of UF compared to women aged 35–40 (OR 2.70, 95%CI: 1.63, 4.47). The odds increased to approximately 4 times among women aged 50–55 (OR 3.72, 95%CI: 2.16, 6.42). The age at menarche after 17 years of age was inversely associated with the presence of UF (OR 0.59, 95%CI: 0.38, 0.92). A late age at last birth was marginally protective against UF among women who gave birth to their last children after the age of 30 (OR 0.65, 95%CI: 0.42, 1.00).

**Table 1:** Characteristics of cases with uterine fibroids and controls and unadjusted odds ratios for uterine fibroids according to selected characteristics.

Variable	Case (n = 335)		Control (n = 539)		Unadjusted OR	
	No.	%	No.	%	OR	95%CI
Age						
35–40	21	6.3	89	16.5	1.00	
40–49	213	63.6	335	62.2	2.70	1.63, 4.47
50–55	101	30.1	115	21.3	3.72	2.16, 6.42
Age at menarche^a^						
≤14	126	37.6	230	42.7	1.00	
15–16	80	23.9	157	29.1	0.93	0.66, 1.32
17+	35	10.4	108	20.0	0.59	0.38, 0.92
Gravidity						
0 pregnancies	3	0.7	4	0.7	1.00	
1 pregnancy	29	8.7	41	7.6	0.94	0.20, 4.54
2 pregnancies	92	27.5	125	23.2	0.98	0.21, 4.49
3 pregnancies	102	30.4	168	31.2	0.81	0.18, 3.69
4 or more pregnancies	109	32.5	201	37.3	0.72	0.16, 3.29
Parity						
0 live births	7	2.1	13	2.4	1.00	
1 live birth	222	66.3	309	57.3	1.33	0.52, 3.40
2 live births	81	24.2	164	30.4	0.92	0.35, 2.39
3 live births	23	6.9	39	7.2	1.10	0.38, 3.14
4 or more live births	2	0.6	14	2.6	0.27	0.05, 1.52
Age at last birth^b^						
<25	95	28.4	137	25.4	1.00	
25–30	150	44.8	233	43.2	0.93	0.67, 1.30
30+	47	14.0	104	19.3	0.65	0.42, 1.00

a Data were available for 71.9% of cases and 91.8% of controls.

b Data were available for 87.2% of cases and 87.9% of controls.

We next evaluated the associations of subclinical atherogenic and cardiovascular risk parameters with UF based on separate logistic regressions (Table 2). For DBP, there was a positive association with UF in the age adjusted and multivariable adjusted models. Women in the highest tertile had multivariate odds of 1.48 (95%CI: 1.01, 2.16, P_trend_ = 0.033) compared to those in the lowest tertile. Elevated ABI was positively associated with the presence of UF in the analysis after controlling for confounding factors. The odds of UF among women in the highest tertile of ABI were 1.51 times higher (95%CI: 1.03, 2.22, P_trend_ = 0.035) relative to those in the lowest tertile. In subgroup for the cardiovascular risk parameters, CRP showed a dose-response pattern with the presence of UF (middle vs. low: OR 1.89, 95%CI: 1.16, 3.09; high vs. low: OR 2.04, 95%CI: 1.21, 3.44; P_trend_ = 0.011). There was an inverse dose-response association between the serum homocysteine and UF with multivariate adjustments (middle vs. low: OR 0.53, 95%CI: 0.34, 0.81; high vs. low: OR 0.49, 95% CI: 0.31, 0.77; P_trend_ = 0.001).

**Table 2:** Characteristics of cases with uterine fibroids and controls and odds ratios of uterine fibroids according to subclinical atherogenic and cardiovascular risk parameters.

	Tertiles	Case (n = 335)	Control (n = 539)	OR1 (95%CI)^a^	OR2 (95%CI)^b^
***Total group***					
BMI, kg/m^2^	1 (<21.94)	83 (24.8)	180 (33.4)	1.00	1.00^c^
	2 (21.94–24.41)	117 (34.9)	181 (33.6)	1.28 (0.89, 1.82)	1.32 (0.92, 1.89)
	3 (>24.41)	135 (40.3)	178 (33.0)	1.36 (0.96, 1.94)	1.40 (0.98, 2.00)
	P_trend_			0.095	0.073
WHR	1 (<0.82)	87 (26.0)	179 (33.2)	1.00	1.00^c^
	2 (0.82–0.87)	132 (39.5)	210 (39.0)	1.16 (0.82, 1.63)	1.17 (0.83, 1.66)
	3 (>0.87)	115 (34.4)	150 (27.8)	1.30 (0.90, 1.87)	1.30 (0.90, 1.88)
	P_trend_			0.161	0.163
TC, mmol/L	1 (>4.47)	108 (32.2)	179 (33.2)	1.00	1.00
	2 (4.47–5.18)	91 (27.2)	181 (33.6)	0.76 (0.53, 1.09)	0.76 (0.53, 1.09)
	3 (>5.18)	136 (40.6)	179 (33.2)	0.95 (0.67, 1.35)	0.92 (0.65, 1.31)
	P_trend_			0.818	0.962
TG, mmol/L	1 (>0.88)	90 (26.9)	174 (32.3)	1.00	1.00
	2 (0.88–1.30)	108 (32.2)	187 (34.7)	0.98 (0.68, 1.39)	0.93 (0.64, 1.34)
	3 (>1.30)	137 (40.9)	178 (33.0)	1.16 (0.82, 1.66)	1.06 (0.73, 1.54)
	P_trend_			0.376	0.716
HDL-C, mmol/L	1 (<1.26)	112 (33.4)	173 (32.1)	1.00	1.00
	2 (1.26–1.56)	137 (40.9)	187 (34.7)	1.20 (0.86, 1.68)	1.27 (0.91, 1.79)
	3 (>1.56)	86 (25.7)	179 (33.2)	0.77 (0.54, 1.10)	0.86 (0.59, 1.25)
	P_trend_			0.176	0.463
LDL-C, mmol/L	1 (>2.66)	103 (30.7)	178 (33.0)	1.00	1.00
	2 (2.66–3.22)	87 (26.0)	183 (34.0)	0.74 (0.51, 1.06)	0.72 (0.50, 1.04)
	3 (>3.22)	145 (43.3)	178 (33.0)	1.08 (0.77, 1.53)	1.03 (0.73, 1.47)
	P_trend_			0.571	0.776
ApoA1, g/L	1 (<1.34)	122 (36.6)	179 (33.2)	1.00	1.00
	2 (1.34–1.52)	119 (35.7)	184 (34.1)	0.88 (0.63, 1.23)	0.94 (0.67, 1.33)
	3 (>1.52)	92 (27.6)	176 (32.7)	0.73 (0.51, 1.03)	0.80 (0.55, 1.14)
	P_trend_			0.074	0.219
ApoB, g/L	1 (<0.72)	98 (29.3)	171 (31.7)	1.00	1.00
	2 (0.72–0.89)	103 (30.7)	191 (35.4)	0.82 (0.57, 1.17)	0.81 (0.57, 1.16)
	3 (>0.89)	134 (40.0)	177 (32.8)	1.00 (0.71, 1.43)	0.92 (0.64, 1.33)
	P_trend_			0.921	0.699
ApoE, mg/dl	1 (<3.09)	106 (31.6)	179 (33.3)	1.00	1.00
	2 (3.09–3.88)	109 (32.5)	183 (34.0)	0.97 (0.69, 1.37)	0.92 (0.65, 1.30)
	3 (>3.88)	120 (35.8)	176 (32.7)	1.06 (0.75, 1.49)	0.96 (0.68, 1.36)
	P_trend_			0.752	0.820
Lipoprotein(a), mg/dl	1 (<16.40)	93 (27.8)	179 (33.2)	1.00	1.00
	2 (16.40–33.80)	127 (37.9)	181 (33.6)	1.26 (0.89, 1.78)	1.31 (0.92, 1.85)
	3 (>33.80)	115 (34.3)	179 (33.2)	1.16 (0.82, 1.64)	1.17 (0.82, 1.67)
	P_trend_			0.427	0.408
Index of dyslipidemia	1 (<0.57)	99 (29.6)	177 (32.8)	1.00	1.00
	2 (0.57–1.03)	108 (32.2)	184 (34.1)	0.93 (0.65, 1.33)	0.87 (0.60, 1.25)
	3 (>1.03)	128 (38.2)	178 (33.0)	1.05 (0.74,1.48)	0.92 (0.64, 1.34)
	P_trend_			0.770	0.696
Index of atherogenicity	1 (<1.77)	80 (23.9)	178 (33.0)	1.00	1.00
	2 (1.77–2.38)	103 (30.7)	180 (33.4)	1.14 (0.79, 1.64)	1.10 (0.76, 1.61)
	3 (>2.38)	152 (45.4)	181 (33.6)	1.51 (1.06, 2.15)	1.39 (0.96, 2.01)
	P_trend_			0.018	0.075
Glucose, mmol/L	1 (<4.85)	87 (26.1)	175 (32.5)	1.00	1.00
	2 (4.85–5.27)	132 (39.6)	185 (34.3)	1.26 (0.89, 1.78)	1.21 (0.85, 1.73)
	3 (>5.27)	114 (34.2)	179 (33.2)	0.96 (0.67, 1.39)	0.91 (0.62, 1.32)
	P_trend_			0.777	0.560
SBP, mmHg	1 (<104)	74 (22.1)	175 (32.5)	1.00	1.00
	2 (104–116)	114 (34.0)	189 (35.1)	1.29 (0.90, 1.87)	1.24 (0.86, 1.80)
	3 (>116)	147 (43.9)	175 (32.5)	1.54 (1.07, 2.22)	1.41 (0.97, 2.06)
	P_trend_			0.022	0.076
DBP, mmHg	1 (<69)	76 (22.7)	165 (30.6)	1.00	1.00
	2 (69–77)	120 (35.8)	215 (39.9)	1.10 (0.77, 1.58)	1.07 (0.74, 1.55)
	3 (>77)	139 (41.5)	159 (29.5)	1.58 (1.10, 2.28)	1.48 (1.01, 2.16)
	P_trend_			0.011	0.033
PWV	1 (<1118.00)	75 (24.1)	170 (33.2)	1.00	1.00
	2 (1118.00–1247.50)	96 (30.9)	172 (33.6)	1.09 (0.74, 1.59)	1.12 (0.76, 1.64)
	3 (>1247.50)	140 (45.0)	170 (33.2)	1.34 (0.91, 1.95)	1.31 (0.89, 1.92)
	P_trend_			0.125	0.159
ABI	1 (<1.04)	73 (23.5)	174 (34.1)	1.00	1.00
	2 (1.04–1.10)	119 (38.3)	185 (36.2)	1.31 (0.91, 1.90)	1.27 (0.88, 1.85)
	3 (>1.10)	119 (38.3)	152 (29.7)	1.50 (1.03, 2.19)	1.51 (1.03, 2.22)
	P_trend_			0.037	0.035
***Subgroup***					
		Case (n = 232)	Control (n = 366)		
CRP, mg/dl	1 (<0.06)	35 (16.5)	97 (29.4)	1.00	1.00
	2 (0.06–0.12)	88 (41.5)	132 (40.0)	1.92 (1.19, 3.11)	1.89 (1.16, 3.09)
	3 (>0.12)	89 (42.0)	101 (30.6)	2.23 (1.37, 3.64)	2.04 (1.21, 3.44)
	P_trend_			0.002	0.011
Hcy, umol/L	1 (<8.90)	100 (43.1)	119 (32.5)	1.00	1.00^d^
	2 (8.90–12.10)	68 (29.3)	127 (34.7)	0.56 (0.37, 0.85)	0.53 (0.34, 0.81)
	3 (>12.10)	64 (27.6)	120 (32.8)	0.54 (0.36, 0.83)	0.49 (0.31, 0.77)
	P_trend_			0.003	0.001
FOL, ng/ml	1 (<8.55)	83 (35.9)	121 (33.2)	1.00	1.00
	2 (8.55–11.27)	74 (32.0)	123 (33.7)	0.84 (0.56, 1.27)	0.86 (0.57, 1.31)
	3 (>11.27)	74 (32.0)	121 (33.2)	0.88 (0.58, 1.33)	0.93 (0.61, 1.41)
	P_trend_			0.537	0.714
Vit B12, pg/ml	1 (<433.57)	83 (35.8)	122 (33.3)	1.00	1.00
	2 (433.57–612.10)	61 (26.3)	122 (33.3)	0.69 (0.45, 1.06)	0.74 (0.48, 1.14)
	3 (>612.10)	88 (37.9)	122 (33.3)	0.93 (0.62, 1.39)	0.97 (0.64, 1.46)
	P_trend_			0.723	0.889

a Age-adjusted.

b Adjusted for age (continuous), BMI (<21.94, 21.94–24.41, >24.41), age at menarche (≤14, 15–16, ≥17, missing), gravity (0, 1, 2, 3, ≥4) and age at last birth (≤24, 25–29, ≥30, missing).

c Adjusted for age (continuous), age at menarche (≤14, 15–16, ≥17, missing), gravity (0, 1, 2, 3, ≥4) and age at last birth (≤24, 25–29, ≥30, missing).

d Adjusted for adjusted for age (continuous), BMI (<21.94, 21.94–24.41, >24.41), age at menarche (≤14, 15–16, ≥17, missing), gravity (0, 1, 2, 3, ≥4), age at last birth (≤24, 25–29, ≥30, missing), folate (<8.55, 8.55–11.27, >11.27) and vitamin B12 (<433.57, 433.57–612.10, >612.10).

Some associations without statistical significance in the overall women were frequently stronger in the hysterectomy-confirmed group (Table 3). We found marginally increased odds of UF with multivariate adjustments (OR 1.87,95% CI: 1.00, 3.49, P_trend_ = 0.056) for BMI. The odds of UF were reduced for HDL-C (OR 0.50, 95% CI: 0.26, 0.94, P_trend_ = 0.048) and ApoA1 (OR 0.49, 95% CI: 0.26, 0.91, P_trend_ = 0.022). The index of atherogenicity showed a positive association with UF (OR 2.20, 95%CI: 1.14, 4.23, P_trend_ = 0.019) in the upper tertile. Regarding the association between cardiovascular risk parameters and UF, higher serum concentration of homocysteine was associated with a significant decrease in the odds of UF (OR 0.45, 95%CI: 0.21, 0.96, P_trend = _0.032) in the upper tertile.

**Table 3:** Characteristics of hysterectomy-confirmed cases with uterine fibroids and controls and odds ratios of uterine fibroids according to subclinical atherogenic and cardiovascular risk parameters.

	Tertiles	Cases (n = 122)	Control (n = 539)	OR1 (95%CI)^a^	OR2 (95%CI )^b^
***Total group***					
BMI, kg/m^2^	1 (<21.94)	23 (18.9)	180 (33.4)	1.00	1.00^c^
	2 (21.94–24.41)	41 (33.6)	181 (33.6)	1.53 (0.86, 2.71)	1.61 (0.85, 3.05)
	3 (>24.41)	58 (47.5)	178 (33.0)	1.73 (0.99, 3.00)	1.87 (1.00, 3.49)
	P_trend_			0.061	0.056
WHR	1 (<0.82)	27 (22.1)	179 (33.2)	1.00	1.00^c^
	2 (0.82–0.87)	47 (38.5)	210 (39.0)	1.21 (0.70, 2.07)	1.11 (0.61, 2.02)
	3 (>0.87)	48 (39.3)	150 (27.8)	1.38 (0.80, 2.40)	1.20 (0.64, 2.24)
	P_trend_			0.251	0.568
TC, mmol/L	1 (>4.47)	35 (28.7)	179 (33.2)	1.00	1.00
	2 (4.47–5.18)	33 (27.0)	181 (33.6)	0.80 (0.46, 1.37)	0.76 (0.41, 1.41)
	3 (>5.18)	54 (44.3)	179 (33.2)	0.89 (0.53, 1.50)	0.79 (0.43, 1.42)
	P_trend_			0.717	0.449
TG, mmol/L	1 (>0.88)	22 (18.0)	174 (32.3)	1.00	1.00
	2 (0.88–1.30)	35 (28.7)	187 (34.7)	1.11 (0.61, 2.02)	0.94 (0.48, 1.84)
	3 (>1.30)	65 (53.3)	178 (33.0)	1.83 (1.05, 3.20)	1.64 (0.87, 3.11)
	P_trend_			0.017	0.072
HDL-C, mmol/L	1 (<1.26)	49 (40.2)	173 (32.1)	1.00	1.00
	2 (1.26–1.56)	50 (41.0)	187 (34.7)	1.05 (0.65, 1.68)	1.04 (0.60, 1.79)
	3 (>1.56)	23 (18.9)	179 (33.2)	0.47 (0.27,0.83)	0.50 (0.26, 0.94)
	P_trend_			0.013	0.048
LDL-C, mmol/L	1 (>2.66)	33 (27.0)	178 (33.0)	1.00	1.00
	2 (2.66–3.22)	30 (24.6)	183 (34.0)	0.69 (0.39, 1.22)	0.66 (0.35, 1.24)
	3 (>3.22)	59 (48.4)	178 (33.0)	1.05 (0.63, 1.76)	1.05 (0.58, 1.89)
	P_trend_			0.681	0.739
ApoA1, g/L	1 (<1.34)	54 (44.6)	179 (33.2)	1.00	1.00
	2 (1.34–1.52)	42 (34.7)	184 (34.1)	0.60 (0.37, 0.98)	0.64 (0.37, 1.14)
	3 (>1.52)	25 (20.7)	176 (32.7)	0.43 (0.25, 0.73)	0.49 (0.26, 0.91)
	P_trend_			0.002	0.022
ApoB, g/L	1 (<0.72)	27 (22.1)	171 (31.7)	1.00	1.00
	2 (0.72–0.89)	38 (31.1)	191 (35.4)	0.90 (0.51, 1.59)	0.98 (0.52, 1.86)
	3 (>0.89)	57 (46.7)	177 (32.8)	1.17 (0.68, 2.02)	1.12 (0.60, 2.10)
	P_trend_			0.478	0.681
ApoE, mg/dl	1 (<3.09)	30 (24.6)	179 (33.3)	1.00	1.00
	2 (3.09–3.88)	42 (34.4)	183 (34.0)	1.40 (0.82, 2.39)	0.99 (0.54, 1.82)
	3 (>3.88)	50 (41.0)	176 (32.7)	1.50 (0.89, 2.53)	0.99 (0.54, 1.79)
	P_trend_			0.139	0.964
Lipoprotein(a), mg/dl	1 (<16.40)	37 (30.3)	179 (33.2)	1.00	1.00
	2 (16.40–33.80)	39 (32.0)	181 (33.6)	0.88 (0.52, 1.49)	0.96 (0.54, 1.73)
	3 (>33.80)	46 (37.7)	179 (33.2)	1.08 (0.65, 1.79)	1.03 (0.58, 1.83)
	P_trend_			0.744	0.923
Index of dyslipidemia	1 (<0.57)	23 (18.9)	177 (32.8)	1.00	1.00
	2 (0.57–1.03)	42 (34.4)	184 (34.1)	1.39 (0.78, 2.47)	1.23 (0.65, 2.37)
	3 (>1.03)	57 (46.7)	178 (33.0)	1.72 (0.99, 2.99)	1.52 (0.81, 2.86)
	P_trend_			0.055	0.190
Index of atherogenicity	1 (<1.77)	19 (15.6)	178 (33.0)	1.00	1.00
	2 (1.77–2.38)	38 (31.1)	180 (33.4)	1.54 (0.84, 2.84)	1.66 (0.84, 3.30)
	3 (>2.38)	65 (53.3)	181 (33.6)	2.23 (1.25, 3.98)	2.20 (1.14, 4.23)
	P_trend_			0.005	0.019
Glucose, mmol/L	1 (<4.85)	29 (24.0)	175 (32.5)	1.00	1.00
	2 (4.85–5.27)	45 (37.2)	185 (34.3)	1.10 (0.64, 1.89)	0.85 (0.46, 1.58)
	3 (>5.27)	47 (38.8)	179 (33.2)	0.91 (0.52, 1.58)	0.77 (0.41, 1.45)
	P_trend_			0.670	0.427
SBP, mmHg	1 (<104)	21 (17.2)	175 (32.5)	1.00	1.00
	2 (104–116)	36 (29.5)	189 (35.1)	1.21 (0.66, 2.21)	0.78 (0.39, 1.56)
	3 (>116)	65 (53.3)	175 (32.5)	1.86 (1.06, 3.28)	1.41 (0.74, 2.70)
	P_trend_			0.020	0.142
DBP, mmHg	1 (<69)	23 (18.9)	165 (30.6)	1.00	1.00
	2 (69–77)	44 (36.1)	215 (39.9)	1.15 (0.65, 2.03)	1.06 (0.55, 2.02)
	3 (>77)	55 (45.1)	159 (29.5)	1.70 (0.97, 2.98)	1.55 (0.82, 2.93)
	P_trend_			0.045	0.137
PWV	1 (<1118.00)	20 (17.9)	170 (33.2)	1.00	1.00
	2 (1118.00–1247.50)	28 (25.0)	172 (33.6)	1.01 (0.53, 1.92)	1.07 (0.53, 2.16)
	3 (>1247.50)	64 (57.1)	170 (33.2)	1.71 (0.95, 3.08)	1.68 (0.87, 3.21)
	P_trend_			0.040	0.084
ABI	1 (<1.04)	27 (24.1)	174 (34.1)	1.00	1.00
	2 (1.04–1.10)	43 (38.4)	185 (36.2)	1.12 (0.64, 1.94)	1.00 (0.54, 1.87)
	3 (>1.10)	42 (37.5)	152 (29.7)	1.11 (0.63, 1.95)	1.45 (0.76, 2.79)
	P_trend_			0.739	0.239
***Subgroup***					
		Cases (n = 95)	Control (n = 366)		
CRP, mg/dl	1 (<0.06)	13 (14.3)	97 (29.4)	1.00	1.00
	2 (0.06–0.12)	33 (36.3)	132 (40.0)	2.00 (0.97, 4.14)	1.67 (0.72, 3.86)
	3 (>0.12)	45 (49.5)	101 (30.6)	2.88 (1.42, 5.84)	1.99 (0.85, 4.69)
	P_trend_			0.003	0.129
Hcy, umol/L	1 (<8.90)	39 (41.1)	119 (32.5)	1.00	1.00^d^
	2 (8.90–12.10)	29 (30.5)	127 (34.7)	0.55 (0.31, 0.99)	0.50 (0.25, 1.01)
	3 (>12.10)	27 (28.4)	120 (32.8)	0.53 (0.30, 0.97)	0.45 (0.21, 0.96)
	P_trend_			0.035	0.032
FOL, ng/ml	1 (<8.55)	36 (37.9)	121 (33.2)	1.00	1.00
	2 (8.55–11.27)	29 (30.5)	123 (33.7)	0.69 (0.39, 1.25)	0.60 (0.29, 1.21)
	3 (>11.27)	30 (31.6)	121 (33.2)	0.82 (0.46, 1.46)	0.69 (0.34, 1.40)
	P_trend_			0.480	0.301
Vit B12, pg/ml	1 (<433.57)	37 (38.9)	122 (33.3)	1.00	1.00
	2 (433.57–612.10)	24 (25.3)	122 (33.3)	0.58 (0.32, 1.07)	0.67 (0.33, 1.38)
	3 (>612.10)	34 (35.8)	122 (33.3)	0.70 (0.39, 1.23)	0.59 (0.29, 1.18)
	P_trend_			0.214	0.130

a Age-adjusted.

b Adjusted for age (continuous), BMI (<21.94, 21.94–24.41, >24.41), age at menarche (≤14, 15–16, ≥17, missing), gravity (0, 1, 2, 3, ≥4) and age at last birth (≤24, 25–29, ≥30, missing).

c Adjusted for age (continuous), age at menarche (≤14, 15–16, ≥17, missing), gravity (0, 1, 2, 3, ≥4) and age at last birth (≤24, 25–29, ≥30, missing).

d Adjusted for adjusted for age (continuous), BMI (<21.94, 21.94–24.41, >24.41), age at menarche (≤14, 15–16, ≥17, missing), gravity (0, 1, 2, 3, ≥4), age at last birth (≤24, 25–29, ≥30, missing), folate (<8.55, 8.55–11.27, >11.27) and vitamin B12 (<433.57, 433.57–612.10, >612.10).

To better demonstrate the individual contribution to UF, we included all associated putative risk factors in complete logistic regressions after adjusting for these risk factors and other potential confounders mutually (Table 4). Consistent with some, but not all, separate analyses we observed a positive effect of ABI (OR 1.88, 95%CI: 1.17, 3.02, P_trend_ = 0.008) on UF, and an inverse dose-response effect of homocysteine (middle vs. low: OR 0.56, 95%CI: 0.36, 0.85; high vs. low: OR 0.50, 95% CI: 0.32, 0.79; P_trend_ = 0.002) in the overall group. There was no evidence of an interaction between ABI and homocysteine (P_interaction_ = 0.903) with UF. In addition, HDL-C (OR 0.46, 95%CI: 0.25, 0.84, P_trend_ = 0.014) was inversely associated with the presence of UF in the hysterectomy-confirmed group.

**Table 4:** Multivariable model of best predictors according to subclinical atherogenic and cardiovascular risk parameters for UF in the overall and hysterectomy-confirmed groups.

	Tertiles	OR^a^	95%CI	P_interaction_
*Ultrasound- or hysterectomy-confirmed cases*				
ABI	1 (<1.04)	1.0		0.903
	2 (1.04–1.10)	1.38	0.90, 2.10	
	3 (>1.10)	1.88	1.17, 3.02	
	P_trend_	0.008		
Hcy, umol/L	1 (<8.90)	1.0		
	2 (8.90–12.10)	0.56	0.36, 0.85	
	3 (>12.10)	0.50	0.32, 0.79	
	P_trend_	0.002		
*Hysterectomy-confirmed cases*				
HDL-C, mmol/L	1 (<1.26)	1.0		–
	2 (1.26–1.56)	0.92	0.53, 1.60	
	3 (>1.56)	0.46	0.25, 0.84	
	P_trend_	0.014		

a Adjusted for age (continuous), BMI (<21.94, 21.94–24.41, >24.41), age at menarche (≤14, 15–16, ≥17, missing), gravity (0, 1, 2, 3, ≥4) and age at last birth (≤24, 25–29, ≥30, missing).

## Discussion

Our study investigated the atherogenic risk factors that were associated with UF for the overall case group. Higher level of ABI was independently associated with increased odds of UF. Conversely, the serum concentration of homocysteine was inversely related to UF. Positive associations were suggested between DBP, serum CRP concentration and UF in separate regressions, but these associations were confounded by other factors. We also analyzed the hysterectomy-confirmed group, an inverse association was observed between HDL-C and UF. Moreover, the effect of BMI, ApoA1, index of atherogenicity and homocysteine on UF was seen in separate regressions, which appeared not significantly when considering other putative risk factors simultaneously.

Nurses’ Health Study II and Black Women’s Health Study, two prospective studies in different ethnic compositions of American women, consistently reported that elevated BMI was associated with an increased risk of UF, and this association was strengthened for hysterectomy-confirmed cases of UF [16], [26], [27]. In two case-control studies, the same pattern between BMI and UF risk was also observed [28], [29]. This association could be explained in two ways. First, obesity is inversely related to the serum level of hormone-binding globulin, resulting in more active circulating estrogens in the body [30]. Excess adipose tissues also contribute to elevated circulating estrogens by converting androgens to estrogens. Second, obesity is associated with atherosclerosis and other atherogenic determinants, including hypertension, hyperinsulinemia and dyslipidemia. These changes in metabolism may stimulate UF cells via mediators such as insulin receptors, insulin-like growth factors, and peroxisome proliferating activating receptors [13]. We found the same trend for a dose-response association between BMI and UF risk, and this effect was marginally significant for women with hysterectomy-confirmed UF in separate analyses. Obesity is important for both UF and atherosclerosis. It is well known that people having a high BMI increases the likelihood of cardiovascular disease and atheroma [31]. Taking full account of the possible effect of obesity, we considered BMI as a confounding variable to determine the associations between other atherogenic risk factors and UF in the present study.

Recent reports have demonstrated that women with hypertension are more likely to be diagnosed with UF [15], [32], [33]. Findings from the Nurses’ Health Study II prospectively revealed that as DBP increased for every 10 mmHg, the risk of UF increased by 8% (95%CI, 5–11%) and 10% (95%CI, 7–13%) in nonusers and users of antihypertensive medications, respectively [25]. Although there was apparently no correlation between blood pressure and UF in the complete regression models, We observed a positive association between DBP and UF using similar analyses as earlier studies [15], [25]. It is possible that elevated blood pressure could cause arterial smooth muscle cell injury and the release of certain cytokines, consequently increasing the risk of developing atheromatous plaques [25], [34]. This process is postulated to be critical to the growth of UF as well [35]. Although it has been suggested that large UF might increase blood pressure by compressing the urinary tract [36], the results of this study support the idea that high blood pressure preceded UF rather than the reverse causality because the positive DBP-UF association were strengthened after women undergo hysterectomy surgery, although the results didn’t reach the statistical significance.

Few studies have investigated an association between lipid metabolism and UF development. Our study results are consistent with an earlier study that demonstrated that there was no association between serum cholesterol levels and UF [37]. It has been reported that women with UF had significantly higher levels of TG level [28]. Such women were also more likely to have higher HDL-C and lower LDL-C levels [38]. However, these studies only compared the lipid parameters directly or on the basis of a small sample size. Because metabolic parameters could be influenced by age, BMI and other potential confounding factors, after adjustment, we found an inverse association between HDL-C and UF in the hysterectomy-confirmed group. ApoA1 is the major protein component of HDL-C in plasma, which has a role in protection against cardiovascular disease associated with HDL-C [31]. Thus, this protective effect of ApoA1 seen in the separate regression might be largely attenuated and remained statistically insignificant after adjustments for HDL-C and other putative risk factors. Moreover, studies that provide information about PWV, ABI, and other atherogenic coefficients were limited. Only Sadlonova et al. [38] have reported that the atherogenic index of women 30–45 years of age with UF was likely to be higher relative to age-matched controls. Our study first reported the positive association between ABI and the presence of UF, but the association was not significant in the hysterectomy-confirmed group. For the index of atherogenicity, women with hysterectomy-confirmed UF had the stronger OR compared to overall cases in the separate regression model, which is consistent with previous finding that the risk of atherosclerosis was increased with a decrease in the mean values of HDL-C/LDL-C in women after surgery on account of UF [39]. As to ApoA1, index of atherogenicity showed a relatively less contribution to UF compared to HDL-C after controlling for all risk factors mutually.

CRP has been used as a marker of inflammation, being a stronger predictor of atherosclerosis [40]. Inflammation is a prominent feature of atherosclerosis [41], and elevation of CRP levels may be an indicate of subclinical atherosclerotic process [42]. Folate and vitamin B12 have biological roles as coenzymes in the synthesis and methylation of DNA, which is critical for preventing cancer development [43]. Deficient folate and vitamin B12 levels can lead to aberrant gene expression and DNA instability and eventually the development of cancer or birth defects [44]. Many studies have reported an inverse association between dietary intake of folate and other B vitamins and the risk of breast cancer [45]. As expected, we observed that serum CRP levels were positively associated with the risk of UF, whereas folate and vitamin B12 levels were inversely associated with UF risk, although the results were not statistically significant.

Homocysteine is an independent risk factor for atherosclerosis [46]. Hyperhomocysteinemia might lead to increased oxidative stress, inhibition of nitric oxide synthase, proliferation of smooth muscle cells, endothelial dysfunction and thrombosis [47]. Mild to moderate elevations in homocysteine concentrations are associated with an increased risk for atherosclerotic vascular diseases [12]. Moreover, hyperhomocysteinemia has been proposed as a risk factor for estrogen-induced tumorigenesis [48]. One case-control study has confirmed that the odds of breast cancer was increased with higher concentrations of homocysteine among pre-menopausal and post-menopausal women [49]. However, we didn’t observe the similar association between homocysteine status and the risk of UF. We favor the idea that the serum concentrations of homocysteine decrease significantly as a result of the progression of a hormonal tumor. It has been reported that homocysteine is required for the development of several types of tumors in animal experiments, and there was an inverse association between tumor size and circulating levels of homocysteine [50]. Our results supported this hypothesis because the inverse association disappeared in women who had these tumors surgically removal. Additional research is needed to elucidate the role of homocysteine concentration in the development of UF.

Our study has some limitations, including that its retrospective nature of a case-control study. We cannot establish causality between exposures and outcomes. Another limitation is the possible selection bias because the data were collected from health examinations. The people undergoing health examinations might have a higher health conscious and a higher likelihood of being diagnosed with UF. However, we included controls who had at least one regular health examination every year for five years to minimize the occasional detection bias. We did not collect information on subjects’ history of smoking or oral contraceptive use, which represents another limitation. These factors might be crucial in a study on the relationship between atherogenic risk factors and UF. In fact, the rates of smoking and oral contraceptive use in a random sample of 100 subjects in our study were very low, thus, we believed that these confounders will not affect our findings.

Our study has several strengths. First, we used ultrasound screening for UF, which enabled us to identify a large sample of subclinical UF cases. The high sensitivity and specificity of ultrasound can avoid misclassification between cases and controls. Second, evidence of atherogenic risk factors such as ABI, serum CRP and homocysteine are reported for the first time in the present study, which provides a new indication to explore the etiology of UF. Third, the present study has a large sample size, which offers information on the epidemiological characteristics of premenopausal women in Chinese, which has been rarely reported in the literature.

In conclusion, the present study demonstrated a positive association between ABI and the presence of UF. Conversely, an inverse association was suggested between HDL-C and UF in the hysterectomy-confirmed group. Unexpectedly, the concentration of homocysteine was inversely related to UF, but this association was not significant in the hysterectomy-confirmed group. These findings suggest that women with UF might have an increased risk of subclinical atherosclerosis. The exact mechanism should be explored in the next step: 1) Further longitudinal studies are needed to replicate these associations using periodic, standardized screening for UF and atherogenic risk factors, data from cohort study would be very valuable; 2) Clinical trails could be conducted to investigate whether the early improvement on atherogenic risk factors, such as obesity, blood pressure and adverse lipid metabolism, reduces the incidence or size of UF and associated complications; and 3) In vitro studies are needed to define the mechanism of action by which different atherogenic risk factors irritate the onset and growth of UF based on the hypothesis that nonhormonal factors also play a role in the development of these tumors.

## References

[1] Farquhar CM, Steiner CA (2002) Hysterectomy rates in the United States 1990–1997. Obstetrics & Gynecology 99: 229–234. 11814502 10.1016/s0029-7844(01)01723-9

[2] Stewart EA (2001) Uterine fibroids. Lancet 357: 293–298. 11214143 10.1016/S0140-6736(00)03622-9

[3] Okolo S (2008) Incidence, aetiology and epidemiology of uterine fibroids. Best Practice & Research in Clinical Obstetrics & Gynaecology 22: 571–588. 10.1016/j.bpobgyn.2008.04.002 18534913

[4] Day Baird D, Dunson DB, Hill MC, Cousins D, Schectman JM (2003) High cumulative incidence of uterine leiomyoma in black and white women: ultrasound evidence. American Journal of Obstetrics & Gynecology 188: 100–107. 12548202 10.1067/mob.2003.99

[5] Buttram VC Jr (1986) Uterine leiomyomata–aetiology, symptomatology and management. Progress in Clinical & Biological Research 225: 275–296. 3538059

[6] Marshall LM, Spiegelman D, Barbieri RL, Goldman MB, Manson JE, et al (1997) Variation in the incidence of uterine leiomyoma among premenopausal women by age and race. Obstetrics & Gynecology 90: 967–973. 9397113 10.1016/s0029-7844(97)00534-6

[7] Parker WH (2007) Etiology, symptomatology, and diagnosis of uterine myomas. Fertility & Sterility 87: 725–736. 17430732 10.1016/j.fertnstert.2007.01.093

[8] Williams VSL, Jones G, Mauskopf J, Spalding J, DuChane J (2006) Uterine fibroids: a review of health-related quality of life assessment. Journal of Women's Health 15: 818–829. 10.1089/jwh.2006.15.818 16999637

[9] Downes E, Sikirica V, Gilabert-Estelles J, Bolge SC, Dodd SL, et al (2010) The burden of uterine fibroids in five European countries. European Journal of Obstetrics, Gynecology, & Reproductive Biology 152: 96–102. 10.1016/j.ejogrb.2010.05.012 20598796

[10] Flake GP, Andersen J, Dixon D (2003) Etiology and pathogenesis of uterine leiomyomas: a review. Environmental Health Perspectives 111: 1037–1054. 12826476 10.1289/ehp.5787 PMC1241553

[11] Walker CL, Stewart EA (2005) Uterine fibroids: the elephant in the room. Science 308: 1589–1592. 15947177 10.1126/science.1112063

[12] Laughlin SK, Schroeder JC, Baird DD (2010) New directions in the epidemiology of uterine fibroids. Seminars in Reproductive Medicine 28: 204–217. 20414843 10.1055/s-0030-1251477 PMC5330647

[13] Schwartz SM, Marshall LM, Baird DD (2000) Epidemiologic contributions to understanding the etiology of uterine leiomyomata. Environmental Health Perspectives 108 Suppl 5821–827. 11035989 10.1289/ehp.00108s5821

[14] Zeigler-Johnson CM, Holmes JL, Lassila HC, Sutton-Tyrrell K, Kuller LH (1998) Subclinical atherosclerosis in relation to hysterectomy status in black women. Stroke 29: 759–764. 9550508 10.1161/01.str.29.4.759

[15] Faerstein E, Szklo M, Rosenshein NB (2001) Risk factors for uterine leiomyoma: a practice-based case-control study. II. Atherogenic risk factors and potential sources of uterine irritation. American Journal of Epidemiology 153: 11–19. 11159140 10.1093/aje/153.1.11

[16] Terry KL, De Vivo I, Hankinson SE, Spiegelman D, Wise LA, et al (2007) Anthropometric characteristics and risk of uterine leiomyoma. Epidemiology 18: 758–763. 17917603 10.1097/EDE.0b013e3181567eed

[17] Danese C, Vestri AR, D'Alfonso V, Deriu G, Dispensa S, et al (2006) Do hypertension and diabetes mellitus influence the site of atherosclerotic plaques? Clinica Terapeutica 157: 9–13. 16669546

[18] Parazzini F, Chiaffarino F, Polverino G, Chiantera V, Surace M, et al (2004) Uterine fibroids risk and history of selected medical conditions linked with female hormones. European Journal of Epidemiology 19: 249–253. 15117118 10.1023/b:ejep.0000020448.43323.2a

[19] Moss NS, Benditt EP (1975) Human atherosclerotic plaque cells and leiomyoma cells. Comparison of in vitro growth characteristics. American Journal of Pathology 78: 175–190. 163592 PMC1913043

[20] van Popele NM, Mattace-Raso FUS, Vliegenthart R, Grobbee DE, Asmar R, et al (2006) Aortic stiffness is associated with atherosclerosis of the coronary arteries in older adults: the Rotterdam Study. Journal of Hypertension 24: 2371–2376. 17082718 10.1097/01.hjh.0000251896.62873.c4

[21] Hasimu B, Li J, Nakayama T, Yu J, Yang J, et al (2006) Ankle brachial index as a marker of atherosclerosis in Chinese patients with high cardiovascular risk. Hypertension Research - Clinical & Experimental 29: 23–28. 10.1291/hypres.29.23 16715650

[22] Ridker PM, Buring JE, Shih J, Matias M, Hennekens CH (1998) Prospective study of C-reactive protein and the risk of future cardiovascular events among apparently healthy women. Circulation 98: 731–733. 9727541 10.1161/01.cir.98.8.731

[23] Hofmann MA, Lalla E, Lu Y, Gleason MR, Wolf BM, et al (2001) Hyperhomocysteinemia enhances vascular inflammation and accelerates atherosclerosis in a murine model. Journal of Clinical Investigation 107: 675–683. 11254667 10.1172/JCI10588 PMC208940

[24] Hamed SA, Nabeshima T (2005) The high atherosclerotic risk among epileptics: the atheroprotective role of multivitamins. Journal of Pharmacological Sciences 98: 340–353. 16079465 10.1254/jphs.crj05003x

[25] Boynton-Jarrett R, Rich-Edwards J, Malspeis S, Missmer SA, Wright R (2005) A prospective study of hypertension and risk of uterine leiomyomata. American Journal of Epidemiology 161: 628–638. 15781952 10.1093/aje/kwi072 PMC4586055

[26] Wise LA, Palmer JR, Spiegelman D, Harlow BL, Stewart EA, et al (2005) Influence of body size and body fat distribution on risk of uterine leiomyomata in U.S. black women. Epidemiology 16: 346–354. 15824551 10.1097/01.ede.0000158742.11877.99 PMC1847589

[27] Marshall LM, Spiegelman D, Manson JE, Goldman MB, Barbieri RL, et al (1998) Risk of uterine leiomyomata among premenopausal women in relation to body size and cigarette smoking. Epidemiology 9: 511–517. 9730029

[28] Takeda T, Sakata M, Isobe A, Miyake A, Nishimoto F, et al (2008) Relationship between metabolic syndrome and uterine leiomyomas: a case-control study. Gynecologic & Obstetric Investigation 66: 14–17. 18230910 10.1159/000114250

[29] Faerstein E, Szklo M, Rosenshein N (2001) Risk factors for uterine leiomyoma: a practice-based case-control study. I. African-American heritage, reproductive history, body size, and smoking. American Journal of Epidemiology 153: 1–10. 11159139 10.1093/aje/153.1.1

[30] Dorgan JF, Reichman ME, Judd JT, Brown C, Longcope C, et al (1995) Relationships of age and reproductive characteristics with plasma estrogens and androgens in premenopausal women. Cancer Epidemiology, Biomarkers & Prevention 4: 381–386. 7655334

[31] Garfagnini A, Devoto G, Rosselli P, Boggiano P, Venturini M (1995) Relationship between HDL-cholesterol and apolipoprotein A1 and the severity of coronary artery disease. Eur Heart J 16: 465–470. 7671890 10.1093/oxfordjournals.eurheartj.a060937

[32] Luoto R, Rutanen EM, Auvinen A (2001) Fibroids and hypertension. A cross-sectional study of women undergoing hysterectomy. Journal of Reproductive Medicine 46: 359–364. 11354837

[33] Templeman C, Marshall SF, Clarke CA, Henderson KD, Largent J, et al (2009) Risk factors for surgically removed fibroids in a large cohort of teachers. Fertility & Sterility 92: 1436–1446. 19019355 10.1016/j.fertnstert.2008.08.074 PMC2765807

[34] Silver MA, Raghuvir R, Fedirko B, Elser D (2005) Systemic hypertension among women with uterine leiomyomata: potential final common pathways of target end-organ remodeling. Journal of Clinical Hypertension 7: 664–668. 16278524 10.1111/j.1524-6175.2005.04384.x PMC8109386

[35] Isobe A, Takeda T, Sakata M, Miyake A, Yamamoto T, et al (2008) Dual repressive effect of angiotensin II-type 1 receptor blocker telmisartan on angiotensin II-induced and estradiol-induced uterine leiomyoma cell proliferation. Human Reproduction 23: 440–446. 17993476 10.1093/humrep/dem247

[36] Hocutt JE Jr (1979) Uterine fibroids and hypertension. Del Med J 51: 697–699. 527739

[37] Ratech H, Stewart ME (1982) Uterine leiomyomas, serum cholesterol, and oral contraceptives. A preliminary study of epidemiologic differences in Los Angeles, California and Albany, New York. Diagnostic Gynecology & Obstetrics 4: 21–24. 7075443

[38] Sadlonova J, Kostal M, Smahelova A, Hendl J, Starkova J, et al (2008) Selected metabolic parameters and the risk for uterine fibroids. International Journal of Gynaecology & Obstetrics 102: 50–54. 18336822 10.1016/j.ijgo.2008.01.022

[39] Kaminski BT, Rzempoluch J, Wiczkowski A, Dyla L, Sobolewska M (1994) Some parameters of lipid metabolism in women 6 years after surgery on account of uterine leiomyoma. Ceska Gynekol 59: 247–250. 7804565

[40] Agrawal A, Hammond DJ Jr, Singh SK (2010) Atherosclerosis-related functions of C-reactive protein. Cardiovascular & Hematological Disorders - Drug Targets 10: 235–240. 20932269 10.2174/187152910793743841 PMC3125067

[41] Libby P, Ridker PM, Hansson GK, Leducq Transatlantic Network on A (2009) Inflammation in atherosclerosis: from pathophysiology to practice. Journal of the American College of Cardiology 54: 2129–2138. 19942084 10.1016/j.jacc.2009.09.009 PMC2834169

[42] Paul A, Ko KWS, Li L, Yechoor V, McCrory MA, et al (2004) C-reactive protein accelerates the progression of atherosclerosis in apolipoprotein E-deficient mice.[Erratum appears in Circulation. 2004 May 11;109(18): 2254]. Circulation 109: 647–655. 14744975 10.1161/01.CIR.0000114526.50618.24

[43] Davis CD, Uthus EO (2004) DNA methylation, cancer susceptibility, and nutrient interactions. Experimental Biology & Medicine 229: 988–995. 15522834 10.1177/153537020422901002

[44] Lin J, Lee IM, Cook NR, Selhub J, Manson JE, et al (2008) Plasma folate, vitamin B-6, vitamin B-12, and risk of breast cancer in women. American Journal of Clinical Nutrition 87: 734–743. 18326613 10.1093/ajcn/87.3.734

[45] Zhang SM, Willett WC, Selhub J, Hunter DJ, Giovannucci EL, et al (2003) Plasma folate, vitamin B6, vitamin B12, homocysteine, and risk of breast cancer. Journal of the National Cancer Institute 95: 373–380. 12618502 10.1093/jnci/95.5.373

[46] Held C, Sumner G, Sheridan P, McQueen M, Smith S, et al (2008) Correlations between plasma homocysteine and folate concentrations and carotid atherosclerosis in high-risk individuals: baseline data from the Homocysteine and Atherosclerosis Reduction Trial (HART). Vascular Medicine 13: 245–253. 18940900 10.1177/1358863X08092102

[47] Borgfeldt C, Andolf E (2000) Transvaginal ultrasonographic findings in the uterus and the endometrium: low prevalence of leiomyoma in a random sample of women age 25–40 years. Acta Obstetricia et Gynecologica Scandinavica 79: 202–207. 10716301

[48] Zhu BT (2003) Medical hypothesis: hyperhomocysteinemia is a risk factor for estrogen-induced hormonal cancer. International Journal of Oncology 22: 499–508. 12579301

[49] Chou Y-C, Lee M-S, Wu M-H, Shih H-L, Yang T, et al (2007) Plasma homocysteine as a metabolic risk factor for breast cancer: findings from a case-control study in Taiwan. Breast Cancer Research & Treatment 101: 199–205. 16850249 10.1007/s10549-006-9278-9

[50] Al-Awadi F, Yang M, Tan Y, Han Q, Li S, et al (2008) Human tumor growth in nude mice is associated with decreased plasma cysteine and homocysteine. Anticancer Research 28: 2541–2544. 19035276

